# Maternal Acrylamide Exposure Modulates Parvalbumin-Positive Interneurons in the Subiculum and Hippocampus of Rat Offspring

**DOI:** 10.3390/biom16050630

**Published:** 2026-04-23

**Authors:** Karol Rycerz, Aleksandra Krawczyk, Ewa Tomaszewska, Piotr Dobrowolski, Siemowit Muszyński, Marcin B. Arciszewski

**Affiliations:** 1 Department of Animal Anatomy and Histology, Faculty of Veterinary Medicine, University of Life Sciences, Akademicka 12, 20-033 Lublin, Poland; karol.rycerz@up.edu.pl (K.R.); mb.arciszewski@wp.pl (M.B.A.); 2 Department of Animal Physiology, Faculty of Veterinary Medicine, University of Life Sciences in Lublin, 20-950 Lublin, Poland; ewa.tomaszewska@up.edu.pl; 3 Department of Functional Anatomy and Cytobiology, Faculty of Biology and Biotechnology, Maria Curie-Sklodowska University, 20-031 Lublin, Poland; piotr.dobrowolski@mail.umcs.pl; 4 Department of Biophysics, Faculty of Environmental Biology, University of Life Sciences in Lublin, 20-950 Lublin, Poland; siemowit.muszynski@up.edu.pl

**Keywords:** acrylamide, hippocampus, parvalbumin, interneurons, weaning, rat

## Abstract

Acrylamide is a neurotoxic compound formed during thermal food processing that can cross the placental barrier and potentially affect fetal brain development. This study aimed to evaluate the effects of maternal acrylamide exposure on parvalbumin-immunoreactive (PV-IR) neurons in the pyramidal layer of the subiculum (Sub) and hippocampus of weaning rats. Pregnant Wistar rats received 3 mg/kg b.w. of acrylamide orally for 5 or 10 days during the prenatal period. After weaning, offspring brains were analyzed using immunohistochemistry, morphometry, and quantitative assessment of PV-IR neuron density and staining intensity in the pyramidal layers of the Sub, Cornu Ammonis 1 (CA1), and Cornu Ammonis 3 (CA3). The results demonstrated a significant increase in PV-IR neuron density in the Sub and CA1 after prolonged maternal exposure, accompanied by a predominance of weakly stained cells and decreased mean immunostaining intensity. Morphometric analyses revealed region-specific changes: enlarged cell area and perimeter with reduced nuclear-to-cytoplasmic ratio in the Sub, whereas CA1 and CA3 showed smaller cell dimensions and altered shapes. In conclusion, maternal acrylamide exposure is associated with region-dependent alterations in the morphology and immunoreactivity of PV-IR neurons within the offspring hippocampus.

## 1. Introduction

Acrylamide is an organic compound formed via the Maillard reaction during the thermal processing of foods rich in carbohydrates and amino acids, especially asparagine [1,2]. Acrylamide is considered a potentially harmful compound to human health and has been classified as a Group 2A probable carcinogen for humans and rodents by the International Agency for Research on Cancer (IARC) and under Commission Regulation (EU) 2017/2158 [3]. Major dietary sources of acrylamide include thermally processed foods such as fried potato products, baked goods, roasted coffee, and smoked plums, which are commonly consumed, including by pregnant women [4]. Additionally, high levels of acrylamide have been detected in poultry feed and other animal feeds based on potatoes and pelleted wheat, as well as in animal products such as eggs and milk [5,6,7]. Many studies indicate the neurotoxic effects of acrylamide on the central and peripheral nervous systems. Moreover, this substance may cross the placental barrier during pregnancy [8,9]. Therefore, acrylamide-induced neurotoxicity may occur both following direct dietary intake in adults and after prenatal exposure resulting from maternal consumption and placental transfer to the developing fetus [3,10,11,12,13]. Numerous studies indicate the role of oxidative stress, neuronal damage, impaired neurotransmission, and neuroinflammatory signaling pathways in the pathophysiology of acrylamide-induced neurotoxicity. Increased levels of reactive oxygen species, malondialdehyde, and DNA damage markers, combined with decreased glutathione levels, suggest a significant role of oxidative stress in nerve cell damage under the influence of acrylamide [1,14].

Since acrylamide is mainly ingested through thermally processed foods, diet may represent an important route of exposure to this compound [1]. At the same time, diet plays a significant role in the development and functioning of the nervous system, particularly in relation to cognitive functions supported by memory-related brain structures [1,15]. The hippocampus proper is a key structure involved in memory and learning processes. It consists of Cornu Ammonis 1–4 (CA1–CA4) areas, which include the polymorphic layer, pyramidal layer, radial layer, and molecular layer. Morphologically, these regions differ by their pyramidal cell density and size, with CA1 containing small, tightly packed neurons, CA2 and CA3 featuring larger, more loosely arranged bodies, and CA4 consisting of scattered cells within the hilar zone [16,17]. A key role in the interaction between the hippocampus and other parts of the brain, especially the entorhinal cortex, is played by the Sub, which has a similar structure to the hippocampus [18]. Due to these connections, it also contributes to memory and learning processes related to spatial orientation [19].

The pyramidal layer contains associated interneuron populations that regulate excitatory–inhibitory balance. These cells are primarily GABAergic interneurons [20,21,22]. Parvalbumin (PV) is expressed in various classes of fast-spiking GABAergic interneurons, but also in some pyramidal neurons of the hippocampus and Sub [23,24]. It is a calcium-binding protein responsible for the transport of calcium ions, the regulation of the activity of various enzymes, and the protection against excessive intracellular concentrations of these ions. Due to their buffering properties, these cells are believed to be less susceptible to neurodegeneration [23,24]. Moreover, the activity of PV-immunoreactive (PV-IR) GABAergic interneurons influences the neurogenesis process and is essential for the development of memory and learning processes in young individuals [25,26]. PV-IR neurons participate in local inhibitory circuits and are widely used as anatomical and molecular markers of fast-spiking GABAergic interneurons. Their distribution and morphology are frequently analyzed to assess structural changes in inhibitory networks in developmental and toxicological studies, although such analyses do not directly indicate functional outcomes [23,24].

Previous studies have shown that acrylamide induces oxidative stress in the brains of fetuses when administered to pregnant mothers and leads to necrosis and hemorrhagic damage to the neuropils [10,27]. Acrylamide may also affect various aspects of hippocampal interneuron function in fetuses and neonates following maternal exposure to this substance. Administration of acrylamide in drinking water to mothers during pregnancy has been shown to increase the density of reelin-producing interneurons in the subgranular zone of the hippocampal dentate gyrus in rats on postnatal day 21, which corresponds to the weaning period. The levels of these cells returned to normal on postnatal day 77 [12]. Maternally exposed offspring showed an increase in the density of GABAergic interneurons expressing glutamic acid decarboxylase 67 following maternal acrylamide administration during pregnancy until post-pregnancy day 21. Changes were noticeable already at a dose of 3.7 mg/kg b.w. [28].

The effect of acrylamide on PV-IR neurons in the hippocampus and Sub has not been investigated to date. The processes related to calcium ion regulation in interneurons under acrylamide exposure remain insufficiently characterized, and their potential relevance to cognitive function requires further study. Therefore, we aimed to determine whether acrylamide administered to mothers during pregnancy may affect the expression of PV in neurons of the Sub and hippocampus, in offspring at weaning. Considering previous studies on changes in interneurons, we hypothesize that maternal acrylamide exposure may alter PV immunoreactivity and morphology in hippocampal interneurons. Because PV is a well-established marker of specific interneuron subtypes, changes in its staining pattern may indicate structural or phenotypic modifications. Hence, the aim of this study was to investigate the immunoreactivity of PV-IR neurons in 21-day-old rats, a critical developmental milestone representing the weaning period, after maternal exposure to acrylamide administered from gestational day 11 or 16 until delivery in the pyramidal layer of the hippocampus and Sub. Specifically, this study sought to assess qualitative and quantitative alterations in the distribution, staining intensity, and morphological characteristics of PV-IR neurons in the Sub, CA1, and CA3 regions.

## 2. Materials and Methods

### 2.1. Animals

All experimental procedures were performed at the Experimental Medicine Center of the Medical University of Lublin, in accordance with the approval of the Local Ethics Committee for Animal Experiments, University of Life Sciences in Lublin, Poland (no. 88/2017). This study was conducted on Wistar rats from 18 mothers. Pregnant females were housed individually in cages, fed with standard laboratory rodent diet (Sniff Spezialdiäten GmbH, Soest, Germany) with access to drinking water ad libitum. Animals were maintained under standard conditions at a constant temperature of 22 ± 1 °C and 55 ± 10% humidity during a 12 h light–dark cycle.

### 2.2. Study Model and Material Collection

The pregnant females were randomly divided into three groups, each containing six mothers. The first group consisted of control animals receiving vehicle (tap water) without acrylamide by oral gavage daily for 10 days. The remaining experimental groups received acrylamide (A8887, Sigma-Aldrich, St. Louis, MO, USA) via oral gavage at a dose of 3 mg/kg b.w. daily. The selected dose is consistent with previous studies on acrylamide toxicity, and it is sufficient to cross the placental barrier and induce measurable oxidative stress in fetuses without causing a significant reduction in litter size and without overt maternal toxicity [29,30]. Although this dose exceeds typical human dietary exposure (0.3–1 µg/kg b.w./day), higher doses are routinely used in rodent studies because acrylamide is metabolized faster in rats and lower doses often fail to produce measurable biological effects relevant to neurodevelopment [31]. Thus, the model is designed to investigate the mechanistic vulnerability of developing hippocampal neurons. Despite the higher dose, the affected pathways, such as oxidative stress and altered interneuron maturation, are conserved across species, providing translational insight into potential neurodevelopmental risks associated with prenatal acrylamide exposure [29,30]. Animals in group I received a vehicle for 5 days. Then, they received acrylamide for 5 days starting from gestational day 16, and those in group II received acrylamide for 10 days (starting from gestational day 11). The two exposure windows (5 days and 10 days) were chosen to target critical periods of hippocampal development: neurogenesis and early differentiation of interneurons, including parvalbumin-positive populations. It has been shown that inhibitory GABAergic interneurons are generated and migrate during mid- to late-embryogenesis in rodents, with much of the interneuron specification occurring between embryonic days 13 and 21 in rats [32]. Females were weighed daily to accurately assess the administered dose. No acrylamide was administered to the rats from birth until weaning. The pups were housed with their mothers and were not further allocated. On day 4 postpartum, some pups were removed to equalize the number of offspring in each group to 8 rats per litter (four males and four females, where possible) to normalize the rearing. On day 21, rats were weaned and anesthetized with an intraperitoneal injection of ketamine and xylazine in NaCl (100 mg/kg + 10 mg/kg, respectively) and euthanized by cervical dislocation according to standard procedures for anesthesia and euthanasia in laboratory animals. Immediately after death, brains were collected and fixed in 4% buffered paraformaldehyde. One rat from each litter was used for the experiment, resulting in six rats per group (control, group I, and group II) to avoid litter effect and pseudo-replication. Thus, each rat constituted an experimental unit for the analysis. To ensure a homogenous experimental group and minimize sex-related physiological variability during early development, only male offspring were selected for further analysis.

### 2.3. Histological Processing and Immunohistochemistry

The collected material was dehydrated, cleared in xylene, and embedded in paraffin blocks using routine histological techniques. The blocks were then cut into 6 µm sections with a microtome and mounted on polysine slides for better adhesion. To inhibit endogenous peroxidase activity, the sections were treated with 0.4% H_2_O_2_ for 30 min at room temperature. To remove unspecific background staining, 10% goat serum (G9023, Sigma Aldrich, St. Louis, MO, USA) was used. The indirect peroxidase–antiperoxidase method was applied to demonstrate PV in nervous cells. The reagents used for the reaction were from Sigma Aldrich (St. Louis, MO, USA) and were diluted in 0.5 M Tris buffer (TBS, pH-7.6), which was also used for rinsing of the slides. The slides were incubated overnight at 4 °C with primary, monoclonal anti-PV antibody produced in mouse (P3088, 1:1000). Subsequently, an anti-mouse secondary antibody against IgG-peroxidase (A9917, 1:200) was used for 1 h at room temperature. To visualize the reaction product, diaminobenzidine (DAB, 32,750) was applied as a chromogen. Additionally, the slides were counterstained with Mayer’s hematoxylin (MHS80). The specificity of the reaction was evaluated by omitting the primary antibody and replacing it with normal serum, which resulted in no reaction product on the slides. The specificity of the anti-parvalbumin antibody (clone PARV-19, P3088) has been described by the manufacturer as selective for parvalbumin, with no cross-reactivity to related EF-hand calcium-binding proteins. Labeling of this antibody clone is abolished after preadsorption with purified PV protein [33]. Slides from the brain cortex were used as a positive control. PV-IR neurons were defined as cells exhibiting clear DAB cytoplasmic staining above background levels, intact soma with a visible counterstained nucleus, and typical interneuron morphology. Cells with ambiguous staining, truncated profiles, or sectioning artifacts were excluded.

### 2.4. Image Acquisition

PV-IR neurons were analyzed and photographed using an Olympus BX51 light microscope (Olympus, Tokyo, Japan). Ten coronal sections per animal (*n* = 6 per group) were selected using systematic random sampling throughout the hippocampal extent. From each section, one image per region (Sub, CA1, CA3) was captured under identical illumination, exposure, and magnification settings. Image selection was based solely on predefined anatomical and technical criteria (complete visibility of the pyramidal layer, lack of staining artifacts, absence of overexposure or saturation, and complete inclusion of the region of interest within the field of view), without reference to staining intensity. This procedure yielded 10 representative images per region per animal, ensuring a robust sampling of the examined brain structures. All image selections were performed blind to the experimental group, and PV-IR neurons in the pyramidal layer of the Sub, CA1, and CA3 of the hippocampus were assessed for their morphology.

### 2.5. PV-IR Neuron Counting and Density Measurement

PV-IR neuron density and total density of all neurons (both PV-IR neurons and negative cells) were counted in a rectangular grid (84 µm × 250 µm; total area, 0.021 mm^2^) overlaid on the image to cover the pyramidal neuron layer. For each hippocampal region, the data obtained from 20 measurements per rat (CA1 and CA3) and 10 measurements per rat (Sub—due to the smaller size of this area) were pooled and averaged for each animal to produce a single animal-level value (the experimental unit) to ensure statistical independence. The cells were counted in ImageJ software (ver.1.54g, National Institutes of Health, Bethesda, MD, USA).

### 2.6. Optical Density (OD) Measurements and Morphometric Analysis

**Figure 1:**
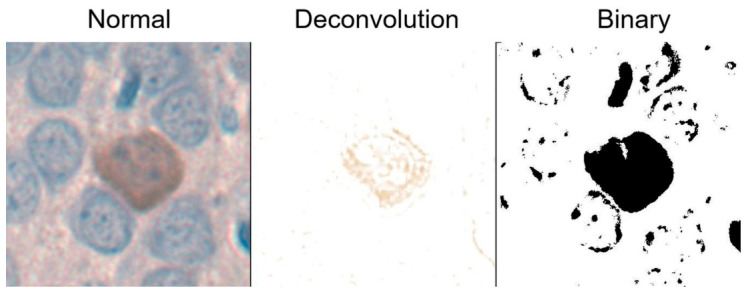
Schematic diagram of image processing in ImageJ software to analyze morphometric parameters of PV-IR neurons.

For further morphometric analyses, color deconvolution and binary image thresholding were applied using an ImageJ software, as demonstrated in Figure 1. Color deconvolution was performed with ImageJ IHC Profiler to provide automatic correction of the background, according to the literature [34]. Eight PV-IR neurons per animal were selected from each region using systematic random sampling (every second PV-IR neuron encountered along a fixed scanning trajectory). This avoided subjective selection and ensured uniform sampling. The eight cell-level measurements were averaged to yield a single value per animal for each parameter.

The immunostaining intensity of PV-IR neurons was analyzed on DAB photomicrographs after deconvolution and converted to 8-bit grayscale images, quantitatively comparing average pixel intensity values based on the ‘average grayscale value’ parameter. The intensity was measured in PV-IR neurons’ cytoplasm manually delineated using a freehand section tool. The grayscale pixel color value ranged from 0 to 255, where 0 corresponded to black (darkest) pixels and 255 was the brightest shade, corresponding to white pixels. These measurements were then converted to optical density (OD) using the formula OD = −log(x/255), where x represents the measured ‘mean gray value’. According to the literature, based on OD, the IHC reaction intensity was classified into the following categories: strong (>0.6), moderate (0.4–0.59), weak (0.2–0.39), and negative (<0.19) [35].

Morphometric analyses, including perimeter, area, shape index (I_s_), and the nuclear–cytoplasmic index (I_N/C_), were evaluated on the same neurons previously selected for intensity analysis. Binary images were used to evaluate the perimeter and area of the neurons, which were automatically delineated using a wand tracing tool. The shape index was calculated using the formula I_s_ = 4πA_C_/P^2^, where A_C_ denotes the cell area and P the cell perimeter. Higher I_s_ values indicate more equilateral or round cell shapes, whereas lower values correspond to more elongated or oval cells. In addition, cell nuclei were manually delineated to determine their surface area and calculate the nuclear–cytoplasmic index (I_N/C_) using a mathematical formula I_N/C_ = A_n_/A_C_, where A_n_ represents the nuclear area and A_C_ the area of perikaryons.

### 2.7. Statistical Analyses

The results were presented as means ± standard deviations, and the semi-quantitative distribution of immunostaining intensity was presented on a jitter plot. Each section of the studied area was individually evaluated and scored in a blinded fashion by one of the authors. Data used for statistical analysis were assumed to be random and independent and measured on an interval scale. Normality of data distribution was assessed using the Shapiro–Wilk test, and homogeneity of variances was evaluated using Levene’s test. Data meeting these assumptions were analyzed using one-way analysis of variance (ANOVA), followed by appropriate post hoc tests. Tukey’s HSD test was applied for all pairwise comparisons among groups, whereas Dunnett’s test was used when experimental groups (groups I and II) were compared specifically to the control group, in order to increase statistical power while controlling the type I error rate. Tukey’s HSD test was used to evaluate the density and immunostaining intensity, and results, including perimeter, area, I_s_, and I_N/C_ from groups I and II, were compared with the control group; therefore, Dunnett’s test was employed. The level of statistical significance of all tests was set at α = 0.05. To prevent pseudo-replication, all section-, image-, and cell-level measurements were first aggregated and averaged within each animal. Statistical tests were performed exclusively on animal-level means (*n* = 6 per group), ensuring that each biological replicate contributed equally to the group mean and variance.

## 3. Results

### 3.1. Microscopic Analyses of PV-IR Neurons

**Figure 2:**
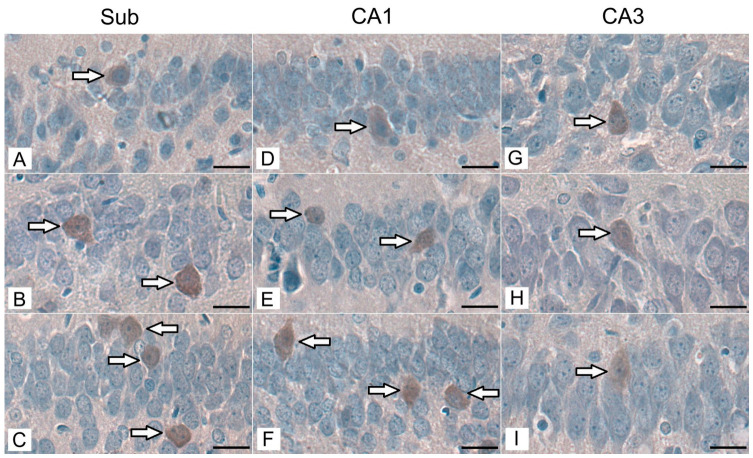
Immunohistochemical staining of PV-IR neurons (arrows) in the pyramidal layer of the Sub, CA1, and CA3 regions in the control group (**A**,**D**,**G**), group I (**B**,**E**,**H**), and group II (**C**,**F**,**I**) of rats. Representative micrographs show PV-IR neuron bodies labeled with DAB in all experimental groups. Scale bar: 20 µm.

Immunohistochemical reactions revealed the presence of PV-IR neurons in the pyramidal layer of the Sub, the CA1 and CA3 regions of the hippocampus proper in control animals, as well as in both groups exposed to acrylamide during the maternal period. PV-IR neurons exhibited brown cytoplasmic immunostaining resulting from the DAB reaction product. The labeled somata were predominantly oval or polygonal in shape, with relatively distinct cell borders and a centrally positioned, round to oval nucleus that appeared pale due to hematoxylin counterstaining. In some cells, short proximal neuronal processes could be distinguished emerging from the soma. PV-IR neurons were distributed singly within the pyramidal layer, intermingled with non-labeled pyramidal neurons. The majority of the analyzed neurons were located in the central part of the pyramidal layer. However, PV-IR neurons were also observed adjacent to both the *stratum moleculare* and *stratum radiatum* in all experimental groups (Figure 2).

### 3.2. Quantitative Analyses of PV-IR Neurons

**Figure 3:**
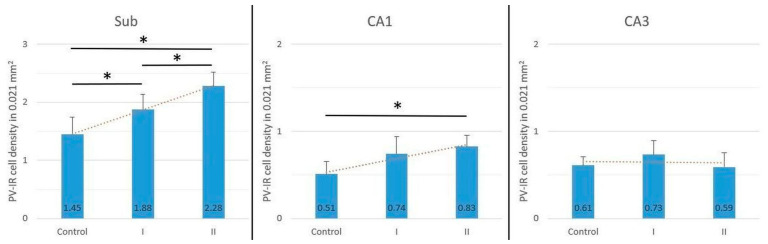
Mean density of PV-IR neurons per 0.021 mm^2^ in the Sub, CA1, and CA3 of the hippocampus in the studied groups of rats (control, group I, and group II). Bars represent mean ± SD. * Statistically significant differences between the studied groups, ANOVA, *p* < 0.05.

In the Sub, the density of PV-IR neurons per 0.021 mm^2^ was significantly higher in animals both after shorter (5 days) and longer (10 days) maternal exposure to acrylamide compared with the control group (ANOVA, *p* < 0.05). A statistically significant difference was also observed between groups I and II of rats (ANOVA, *p* < 0.05), indicating time-dependent changes. In CA1, there was a significant increase in PV-IR neuron density observed only in animals from group II (ANOVA, *p* < 0.05), while an increase in group I was not statistically significant (ANOVA, *p* > 0.05). In CA3, no statistically significant differences in PV-IR neuron density were observed among the studied groups (ANOVA, *p* > 0.05) (Figure 3). Total density of all cells (PV-IR neurons + PV-negative cells) across all experimental groups and hippocampal regions (Sub, CA1, and CA3) did not reach statistical significance.

**Figure 4:**
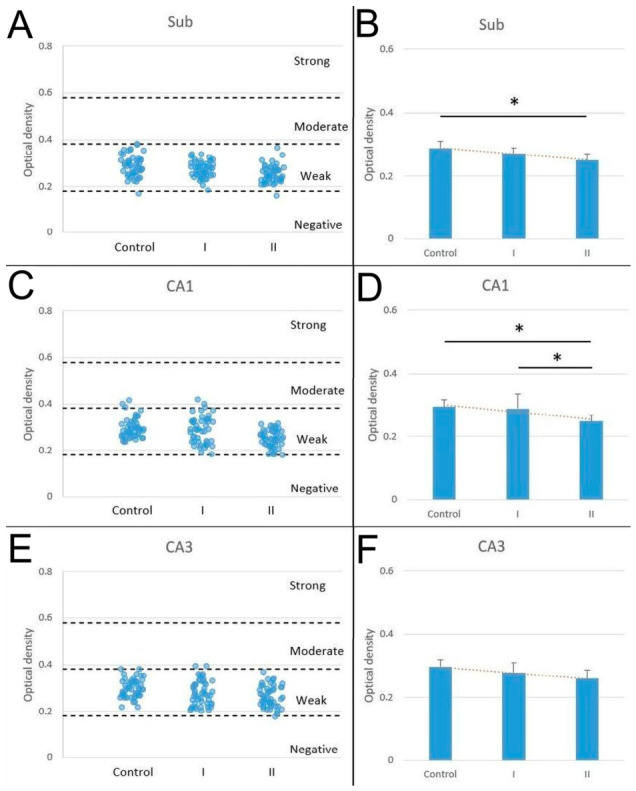
Distribution of PV-IR neurons classified according to staining intensity (strong, moderate, weak, and negative) in the Sub (**A**), CA1 (**C**), and CA3 (**E**) in the control group, group I, and group II of rats (48 cells analyzed per group). Mean optical density of PV-IR neurons in the Sub (**B**), CA1 (**D**), and CA3 (**F**) in the studied groups of rats (control, group I, and group II), where a rat constitutes an experimental unit. Bars represent mean ± SD. * statistically significant differences between the studied groups, ANOVA, *p* < 0.05.

Most PV-IR neurons exhibited weak immunoreactivity across all studied regions and experimental groups. No strongly stained neurons were observed. In the Sub, weakly stained cells accounted for 97.92% in the control group, 100% in group I, and 97.92% in group II (Figure 4A). In CA1, the majority of PV-IR neurons were weakly stained in all groups, with percentages of 93.75% in the control group, 93.75% in group I, and 100% in group II. The remaining cells exhibited moderate staining. Group II showed a higher percentage of weakly stained neurons compared with the control group and group I (Figure 4C). In CA3, PV-IR neurons were predominantly weakly stained in all experimental groups (Figure 4E). Since the majority of PV-IR neurons exhibited weak immunostaining, the differences between the experimental groups were further evaluated by analyzing the mean reaction intensity expressed as optical density (OD). This quantitative analysis revealed significant statistical differences between the groups of animals. A downward trend was observed in animals after maternal exposure to acrylamide. The lowest mean intensity of the reaction was observed in neurons of animals from mothers exposed to acrylamide for a longer time (10 days) in comparison to the control group. These results were demonstrated within the pyramidal layer in the Sub and CA1, and the differences were statistically significant (ANOVA, *p* < 0.05), whereas in CA3, the decrease was statistically insignificant (Figure 4B,D,E).

Analysis of cell size and shape revealed no prominent differences among the experimental groups, although some trends were observed. In the Sub, PV-IR neuron perimeter and area values were higher, particularly in group II (Dunnett, *p* < 0.05, perimeter; Dunnett, *p* < 0.05, area). Correspondingly, the I_N/C_ was decreased in group II, reflecting an increase in cytoplasmic area but without statistical significance. In the Sub, PV-IR neurons showed lower I_s_ values in group I, indicating a more elongated cell outline (ANOVA, *p* < 0.05) (Table 1, Sub). In CA1, PV-IR neuron perimeter and area were decreased in comparison to the control group, showing statistical significance in both I and II groups (Dunnett, *p* < 0.05, perimeter; Dunnett, *p* < 0.05, area). Consequently, I_N/C_ was significantly increased, reflecting a relative increase in nuclear area, in both group I and group II compared with controls (Dunnett, *p* < 0.05). According to I_s_, neurons did not show any statistical differences (Table 1, CA1). In CA3, PV-IR neuron perimeter was decreased in both experimental groups compared with controls, but with statistical significance only in group II. Cell area was significantly decreased, showing a statistical difference only in group I (ANOVA, *p* < 0.05). An increase in I_N/C_ was demonstrated in the group of animals that received acrylamide for a longer time (10 days). There were no statistically significant differences in I_s_ between acrylamide-exposed and control animals (Table 1, CA3).

## 4. Discussion

Maternal exposure to acrylamide induced distinct changes in PV-IR neurons in the hippocampal formation of offspring. PV-IR neurons were present in all examined regions, with a significant increase in density observed in the Sub and CA1 after prolonged exposure. Most PV-IR neurons exhibited weak staining, accompanied by a decrease in mean optical density, indicating reduced PV signal in exposed animals. Morphometric analyses revealed region-specific differences, with enlarged PV-IR neurons in the Sub and smaller ones in CA1 and CA3. These findings indicate that maternal acrylamide exposure leads to changes in the number and morphology of PV-IR neurons in the hippocampus.

To date, no studies have investigated the effects of acrylamide on PV in the central nervous system. However, it has been shown that PV is predominantly expressed in fast spiking GABAergic interneurons [23,24]. Thus, the observed increase in the density of PV-IR neurons, particularly in the Sub and in CA1 regions following prolonged maternal exposure to acrylamide, is consistent with previous studies on the effects of acrylamide on GABAergic interneurons. It has been demonstrated that acrylamide, administered by oral gavage at doses of 5, 15, and 30 mg/kg b.w. for five consecutive days, induces a dose-dependent increase in the density of GABAergic interneurons and expression of GAD-67 in the cerebellum of weaning rats [36]. Other studies have reported increased densities of reelin-positive interneurons without affecting the overall GABAergic population, suggesting a structural response to altered neurogenesis, even at the lowest acrylamide doses [28]. Similar studies were conducted in weaning pups from dams receiving acrylamide in drinking water from day 10 of pregnancy to postnatal day 21 at doses of 4, 20, and 100 ppm. The results indicated an increased density of reelin-positive interneurons in the dentate gyrus at postnatal day 21 following exposure to 100 ppm acrylamide. However, in animals assessed at postnatal day 77, no changes in the density of these cells were observed, suggesting a weak and reversible effect of acrylamide [12]. Considering previous reports and our own results, the elevated PV-IR neuron density observed after acrylamide exposure likely reflects structural modifications in PV immunoreactivity. While acrylamide has been associated with oxidative and calcium-related disturbances in other models [14,15,16,17,18,19,20,21,22,23,24,25,26,27], the present findings indicate solely changes at the level of PV staining patterns, without providing evidence of functional or mechanistic implications. Furthermore, the neurotoxic effects of acrylamide may lead to the loss of various neuronal populations, as well as to increased expression of this protein in neurons that were previously non-reactive, thereby relatively increasing their density [37,38]. It has been shown that administration of acrylamide to six-week-old rats at doses of 5, 10, and 20 mg/kg body weight results in a trend toward increased density of PV-IR neurons, although these changes are not statistically significant [39]. However, it has been shown that acrylamide reduces the number of progenitor cells and nearly mature neurons, as well as disrupts their outgrowth and maturation. The compound significantly reduces the proliferation of neural progenitor cells, inducing apoptosis and cell death [39,40]. Therefore, acrylamide may affect some of the PV-IR neuron populations that are known to interact with neurogenic niches [25].

In contrast, our results showed a decrease in PV immunostaining intensity of PV-IR neurons in the Sub and CA1 from rats whose mothers received acrylamide during pregnancy for a period of 10 days. To date, the effect of acrylamide on PV protein expression in neurons has not been investigated. Therefore, it is difficult to explain this phenomenon. The trend of PV downregulation is demonstrated in all examined areas, visible both in the semi-quantitative jitter plot (although the vast majority of observations exhibited weak immunostaining, distinct differences remained discernible within this intensity range) and optical density measurements (Figure 4). However, it should be noted that both approaches have inherent limitations: the categorical classification used in the jitter plot represents a semi-quantitative assessment, while optical density measurements may be influenced by technical factors such as staining conditions, image acquisition, and processing parameters, and should therefore be interpreted with appropriate caution. The results of immunostaining intensity suggest that the investigated substance may affect certain aspects of the calcium-binding protein immunoreactivity, which should be supported by further studies on PV expression or its mRNA. Acrylamide has been shown to induce oxidative stress and disrupt calcium regulation and mitochondrial integrity [41], which may contribute to the altered PV immunoreactivity observed in our study, reflecting changes in protein detectability or expression rather than functional consequences. Decreased PV staining intensity has also been reported in other experimental conditions, such as in the insular cortex of Shank3 knockout mice, illustrating that PV immunoreactivity can vary independently of interneuron number [42]. The other authors indicate a shift in the balance between excitation and inhibition toward increased inhibition, pointing to the regulatory role of PV in neurotransmission and interneuron plasticity in centers responsible for memory, such as the hippocampus and the striatum [43,44]. However, without molecular or physiological assays, the present findings should be interpreted strictly as structural changes in PV labeling, and the underlying cellular mechanisms remain undetermined.

Our own results on the maternal impact of acrylamide on PV-IR neurons in the pyramidal layer of the Sub and hippocampal CA1 and CA3 regions of weaning rats indicate an interesting relationship, in which an increase in the density of PV-IR neurons is accompanied by a decrease in their staining intensity, depending on the duration of exposure to the investigated substance. This suggests that staining intensity does not necessarily reflect neuron number. Reduced PV immunoreactivity has been shown to reflect decreased intracellular PV protein levels rather than interneuron loss [45]. Since the total density of PV-IR neurons and PV-negative cells showed no statistically significant differences, we can likely exclude the possibility that the relative increase in the PV-IR neuron proportion resulted from the loss of other neuronal populations. Some authors suggest that GABAergic interneurons are more resistant to acrylamide neurotoxicity than other neuronal types [46], and increases in their density may represent compensatory responses to disrupted calcium homeostasis [27,28,41]. Additionally, oxidative stress and axonal transport disturbances may alter protein expression, including PV [47]. The apparent increase in PV-IR neuron density may also reflect altered antigen detectability rather than true changes in neuron number, as PV immunoreactivity is sensitive to epitope accessibility and cellular stress [48,49,50]. Technical factors such as variability in staining efficiency or antibody penetration, as well as biological factors, including changes in PV protein expression per cell, may contribute to the observed pattern. Another possible explanation is stress-related phenotypic remodeling, in which neurons with previously low or undetectable PV levels become weakly immunopositive. Similar shifts in interneuron marker expression have been reported under metabolic or neurotoxic stress [51]. This interpretation is consistent with the lack of changes in total cell counts. Reduced staining intensity may therefore correspond to lower detectable PV content per cell, as described in other models, including Shank3-deficient mice [42]. Finally, the observed pattern may also reflect shifts in classification thresholds, as immunohistochemical categorization depends on staining intensity distribution, which can influence cell counts near threshold values [51,52].

The region-specific morphological changes in PV-IR neurons observed in our study following prenatal exposure to acrylamide indicate a differential susceptibility of the Sub, CA1, and CA3 regions to the effects of this compound. In the Sub, an increase in the area and perimeter of PV-IR neurons may suggest adaptive processes or reactive cytoplasmic hypertrophy. This phenomenon is consistent with reported mechanisms of acrylamide neurotoxicity, including the induction of oxidative stress, cytoskeletal damage, and disruption of axonal transport, which lead to cytoplasmic swelling or changes in its protein composition [41]. Notably, the PV population in the Sub is heterogeneous and includes glutamatergic neurons, which may respond differently to toxic factors than classical GABAergic interneurons [53]. In contrast, in the CA1 and CA3 regions, features of reduced cell dimensions (decrease in perimeter/area) predominated, which may indicate trophic deficits or inhibition of morphological maturation. Acrylamide has been shown to affect neuronal development, including neurite growth and hippocampal neurogenesis, which may underlie these region-specific changes [54]. Altered I_N/C_ values in CA1 and CA3 may reflect both the reduction in cytoplasmic volume and the disruption of nuclear–cytoplasmic homeostasis, which was described in neurons exposed to severe oxidative stress [47]. PV interneurons are considered particularly sensitive to metabolic disturbances and oxidative stress due to their high energy demands and strong dependence on proper Ca^2+^ regulation, which may explain their markedly altered morphology in CA1 and CA3, as well as the differential response observed in the Sub [47,55]. These patterns likely reflect the intrinsic anatomical and developmental heterogeneity of hippocampal subfields. Reduced PV-IR neuron dimensions observed in CA1 and CA3, contrasting with increased size in the Sub, may reflect differences in cytoarchitecture and maturation dynamics. The CA1 region, characterized by densely packed neurons, is especially vulnerable to metabolic and oxidative disturbances [56], consistent with the reduced cell size and increased I_N/C_ ratio observed here. In contrast, the subiculum, as a transitional zone between the hippocampus proper and cortical areas, exhibits greater structural heterogeneity and distinct neuronal organization [57], which may underlie the increased PV-IR neuron size following acrylamide exposure. Phenotypically diverse PV-IR neurons in the subiculum, including non-classical cell types, may contribute to the distinct morphological changes observed in this region [57]. Given that PV-IR neurons exhibit varying metabolic demand and sensitivity to calcium dysregulation across different hippocampal regions, acrylamide-induced oxidative stress may differentially affect their morphology depending on the local cellular environment [56,57,58]. Taken together, these anatomical and phenotypic differences may explain the differential structural response of PV-IR neurons to maternal acrylamide exposure.

## 5. Conclusions

To conclude, maternal exposure to acrylamide induced clear, region-specific alterations in PV-IR neurons within the offspring hippocampus. Regarding our hypothesis, the most pronounced effects were observed in the Sub and CA1, where a significant increase in PV-IR neuron density was found after prolonged exposure, while CA3 showed only non-significant trends. Despite these quantitative increases, acrylamide exposure consistently led to a marked reduction in PV immunostaining intensity across all hippocampal subregions, accompanied by a higher proportion of weakly stained neurons. This observation may suggest a possible reduction in parvalbumin immunoreactivity due to changes in PV detectability or expression. Morphometric analyses revealed subtle but region-dependent changes in PV-IR neuronal structure. Enlarged cell size and decreased I_N/C_ ratio in the Sub contrasted with reduced size and increased I_N/C_ ratio in CA1, while CA3 showed only minor alterations. These findings indicate heterogeneous susceptibility of hippocampal interneurons to acrylamide. The present findings, therefore, indicate structural and immunohistochemical modifications of PV-IR neuron populations following prenatal acrylamide exposure. Further molecular and electrophysiological studies are required to clarify the functional implications of maternal acrylamide exposure in PV-IR neurons in weaning rats.

## Figures and Tables

**Table 1:** Morphometric parameters in PV-IR neurons, including perimeter (µm), area (µm^2^), shape index, and nuclear–cytoplasmic ratio in the Sub, CA1, and CA3 hippocampal areas of studied rats from control (Cont.), I, and II groups. Data shows mean values with standard deviation. * Statistically significant differences, Dunnett, *p* < 0.05, in reference to the control group.

	Sub			CA1			CA3		
	Cont.	I	II	Cont.	I	II	Cont.	I	II
Perimeter (µm)	71.82 ± 6.99	91.77 * ± 11.67	93.52 * ± 19.62	82.93 ± 4.29	69.97 * ± 10.02	70.21 * ± 4.09	98.19 ± 11.89	85.48 ± 12.09	77.98 * ± 13.13
Area (µm^2^)	150.04 ± 12.47	133.69 ± 14.12	180.93 * ± 24.18	159.68 ± 10.27	133.47 * ± 8.54	143.41 * ± 14.33	172.79 ± 10.74	127.03 * ± 6.27	165.83 ± 16.16
Shape Index	0.41 ± 0.03	0.26 * ± 0.08	0.32 ± 0.11	0.32 ± 0.02	0.38 ± 0.06	0.31 ± 0.06	0.32 ± 0.07	0.26 ± 0.04	0.39 ± 0.09
Nuclear-cytoplasmic index	0.45 ± 0.03	0.46 ± 0.04	0.40 ± 0.02	0.38 ± 0.02	0.47 * ± 0.02	0.50 * ± 0.01	0.46 ± 0.03	0.49 ± 0.02	0.51 * ± 0.03

## Data Availability

The original contributions presented in this study are included in the article. Further inquiries can be directed to the corresponding author.

## Abbreviations

The following abbreviations are used in this manuscript:

PV	Parvalbumin
PV-IR	Parvalbumin immunoreactive
GABA	Gamma-aminobutyric acid
GAD-67	Glutamic acid decarboxylase 67
Ca^2+^	Calcium ions
Sub	Subiculum
CA	Cornu Ammonis
CA1	Cornu Ammonis 1 (hippocampal field CA1)
CA3	Cornu Ammonis 3 (hippocampal field CA3)
DAB	Diaminobenzidine
TBS	Tris-buffered saline
OD	Optical density
I_s_	Shape index
I_N/C_	Nuclear-to-cytoplasmic ratio index
ANOVA	Analysis of variance
HSD	Honestly significant difference
IARC	International Agency for Research on Cancer
EFSA	European Food Safety Authority
